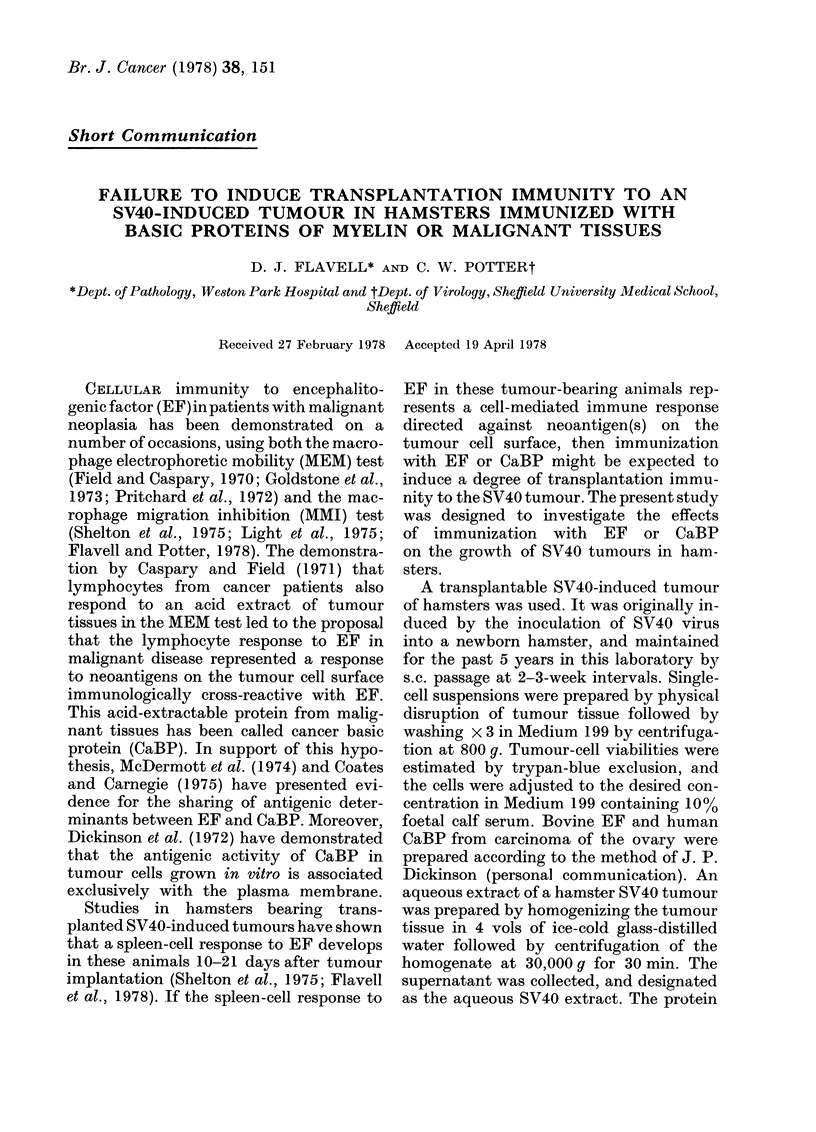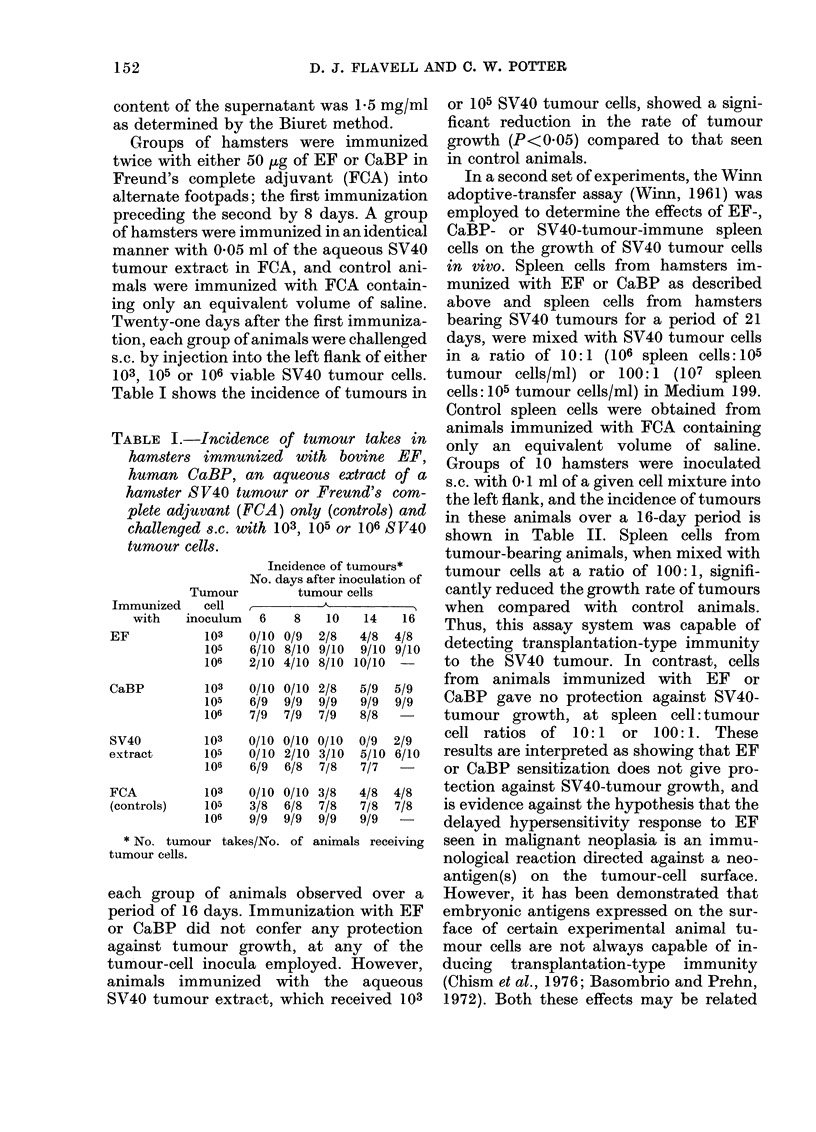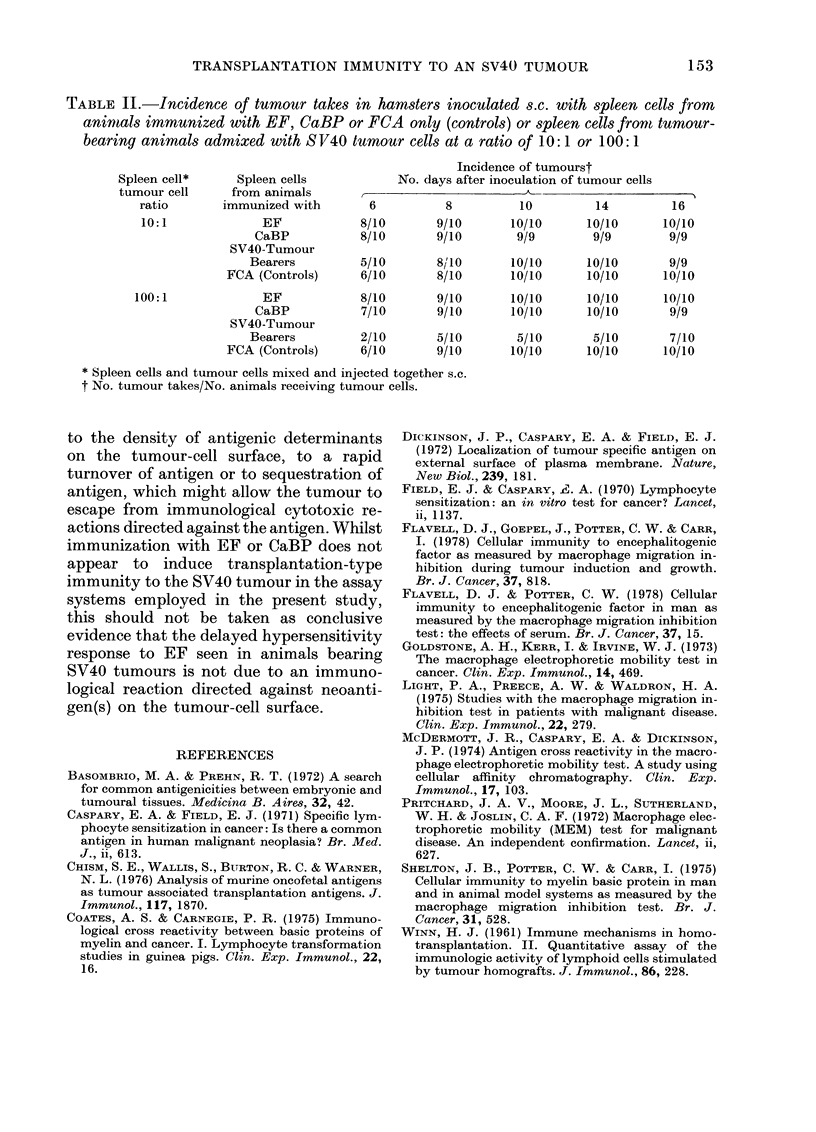# Failure to induce transplantation immunity to an SV40-induced tumour in hamsters immunized with basic proteins of myelin or malignant tissues.

**DOI:** 10.1038/bjc.1978.176

**Published:** 1978-07

**Authors:** D. J. Flavell, C. W. Potter


Br. J. Cancer (1978) 38, 151

### *Short Communication*

### FAILURE TO INDUCE TRANSPLANTATION IMMUNITY TO AN SV40-INDUCED TUMOUR IN HAMSTERS IMMUNIZED WITH BASIC PROTEINS OF MYELIN OR MALIGNANT TISSUES

D. J. FLAVELL* AND C. W. POTTER†

*Dept. of Pathology, Weston Park Hospital and †Dept. of Virology, Sheffield University Medical School, Sheffield

Received 27 February 1978 Accepted 19 April 1978

CELLULAR immunity to encephalitogenic factor (EF) in patients with malignant neoplasia has been demonstrated on a number of occasions, using both the macrophage electrophoretic mobility (MEM) test (Field and Caspary, 1970; Goldstone *et al.*, 1973; Pritchard *et al.*, 1972) and the macrophage migration inhibition (MMI) test (Shelton *et al.*, 1975; Light *et al.*, 1975; Flavell and Potter, 1978). The demonstration by Caspary and Field (1971) that lymphocytes from cancer patients also respond to an acid extract of tumour tissues in the MEM test led to the proposal that the lymphocyte response to EF in malignant disease represented a response to neoantigens on the tumour cell surface immunologically cross-reactive with EF. This acid-extractable protein from malignant tissues has been called cancer basic protein (CaBP). In support of this hypothesis, McDermott *et al.* (1974) and Coates and Carnegie (1975) have presented evidence for the sharing of antigenic determinants between EF and CaBP. Moreover, Dickinson *et al.* (1972) have demonstrated that the antigenic activity of CaBP in tumour cells grown *in vitro* is associated exclusively with the plasma membrane.

Studies in hamsters bearing transplanted SV40-induced tumours have shown that a spleen-cell response to EF develops in these animals 10–21 days after tumour implantation (Shelton *et al.*, 1975; Flavell *et al.*, 1978). If the spleen-cell response to EF in these tumour-bearing animals represents a cell-mediated immune response directed against neoantigen(s) on the tumour cell surface, then immunization with EF or CaBP might be expected to induce a degree of transplantation immunity to the SV40 tumour. The present study was designed to investigate the effects of immunization with EF or CaBP on the growth of SV40 tumours in hamsters.

A transplantable SV40-induced tumour of hamsters was used. It was originally induced by the inoculation of SV40 virus into a newborn hamster, and maintained for the past 5 years in this laboratory by s.c. passage at 2–3-week intervals. Single-cell suspensions were prepared by physical disruption of tumour tissue followed by washing $\times 3$ in Medium 199 by centrifugation at 800 $g$. Tumour-cell viabilities were estimated by trypan-blue exclusion, and the cells were adjusted to the desired concentration in Medium 199 containing 10% foetal calf serum. Bovine EF and human CaBP from carcinoma of the ovary were prepared according to the method of J. P. Dickinson (personal communication). An aqueous extract of a hamster SV40 tumour was prepared by homogenizing the tumour tissue in 4 vols of ice-cold glass-distilled water followed by centrifugation of the homogenate at 30,000 $g$ for 30 min. The supernatant was collected, and designated as the aqueous SV40 extract. The protein content of the supernatant was 1·5 mg/ml as determined by the Biuret method.

Groups of hamsters were immunized twice with either 50 μg of EF or CaBP in Freund's complete adjuvant (FCA) into alternate footpads; the first immunization preceding the second by 8 days. A group of hamsters were immunized in an identical manner with 0·05 ml of the aqueous SV40 tumour extract in FCA, and control animals were immunized with FCA containing only an equivalent volume of saline. Twenty-one days after the first immunization, each group of animals were challenged s.c. by injection into the left flank of either $10^3$, $10^5$ or $10^6$ viable SV40 tumour cells. Table I shows the incidence of tumours in each group of animals observed over a period of 16 days. Immunization with EF or CaBP did not confer any protection against tumour growth, at any of the tumour-cell inocula employed. However, animals immunized with the aqueous SV40 tumour extract, which received $10^3$ or $10^5$ SV40 tumour cells, showed a significant reduction in the rate of tumour growth ($P<0·05$) compared to that seen in control animals.

TABLE I.—*Incidence of tumour takes in hamsters immunized with bovine EF, human CaBP, an aqueous extract of a hamster SV40 tumour or Freund's complete adjuvant (FCA) only (controls) and challenged s.c. with* $10^3$, $10^5$ *or* $10^6$ *SV40 tumour cells.*

| Immunized with | Tumour cell inoculum | Incidence of tumours* No. days after inoculation of tumour cells: 6 | 8 | 10 | 14 | 16 |
|---|---|---|---|---|---|---|
| EF | $10^3$ | 0/10 | 0/9 | 2/8 | 4/8 | 4/8 |
| | $10^5$ | 6/10 | 8/10 | 9/10 | 9/10 | 9/10 |
| | $10^6$ | 2/10 | 4/10 | 8/10 | 10/10 | — |
| CaBP | $10^3$ | 0/10 | 0/10 | 2/8 | 5/9 | 5/9 |
| | $10^5$ | 6/9 | 9/9 | 9/9 | 9/9 | 9/9 |
| | $10^6$ | 7/9 | 7/9 | 7/9 | 8/8 | — |
| SV40 extract | $10^3$ | 0/10 | 0/10 | 0/10 | 0/9 | 2/9 |
| | $10^5$ | 0/10 | 2/10 | 3/10 | 5/10 | 6/10 |
| | $10^6$ | 6/9 | 6/8 | 7/8 | 7/7 | — |
| FCA (controls) | $10^3$ | 0/10 | 0/10 | 3/8 | 4/8 | 4/8 |
| | $10^5$ | 3/8 | 6/8 | 7/8 | 7/8 | 7/8 |
| | $10^6$ | 9/9 | 9/9 | 9/9 | 9/9 | — |

* No. tumour takes/No. of animals receiving tumour cells.

In a second set of experiments, the Winn adoptive-transfer assay (Winn, 1961) was employed to determine the effects of EF-, CaBP- or SV40-tumour-immune spleen cells on the growth of SV40 tumour cells *in vivo*. Spleen cells from hamsters immunized with EF or CaBP as described above and spleen cells from hamsters bearing SV40 tumours for a period of 21 days, were mixed with SV40 tumour cells in a ratio of 10:1 ($10^6$ spleen cells:$10^5$ tumour cells/ml) or 100:1 ($10^7$ spleen cells:$10^5$ tumour cells/ml) in Medium 199. Control spleen cells were obtained from animals immunized with FCA containing only an equivalent volume of saline. Groups of 10 hamsters were inoculated s.c. with 0·1 ml of a given cell mixture into the left flank, and the incidence of tumours in these animals over a 16-day period is shown in Table II. Spleen cells from tumour-bearing animals, when mixed with tumour cells at a ratio of 100:1, significantly reduced the growth rate of tumours when compared with control animals. Thus, this assay system was capable of detecting transplantation-type immunity to the SV40 tumour. In contrast, cells from animals immunized with EF or CaBP gave no protection against SV40-tumour growth, at spleen cell:tumour cell ratios of 10:1 or 100:1. These results are interpreted as showing that EF or CaBP sensitization does not give protection against SV40-tumour growth, and is evidence against the hypothesis that the delayed hypersensitivity response to EF seen in malignant neoplasia is an immunological reaction directed against a neoantigen(s) on the tumour-cell surface. However, it has been demonstrated that embryonic antigens expressed on the surface of certain experimental animal tumour cells are not always capable of inducing transplantation-type immunity (Chism *et al.*, 1976; Basombrio and Prehn, 1972). Both these effects may be related

TABLE II.—*Incidence of tumour takes in hamsters inoculated s.c. with spleen cells from animals immunized with EF, CaBP or FCA only (controls) or spleen cells from tumour-bearing animals admixed with SV40 tumour cells at a ratio of* 10:1 *or* 100:1

| Spleen cell* tumour cell ratio | Spleen cells from animals immunized with | Incidence of tumours† No. days after inoculation of tumour cells 6 | 8 | 10 | 14 | 16 |
|---|---|---|---|---|---|---|
| 10:1 | EF | 8/10 | 9/10 | 10/10 | 10/10 | 10/10 |
| | CaBP | 8/10 | 9/10 | 9/9 | 9/9 | 9/9 |
| | SV40-Tumour Bearers | 5/10 | 8/10 | 10/10 | 10/10 | 9/9 |
| | FCA (Controls) | 6/10 | 8/10 | 10/10 | 10/10 | 10/10 |
| 100:1 | EF | 8/10 | 9/10 | 10/10 | 10/10 | 10/10 |
| | CaBP | 7/10 | 9/10 | 10/10 | 10/10 | 9/9 |
| | SV40-Tumour Bearers | 2/10 | 5/10 | 5/10 | 5/10 | 7/10 |
| | FCA (Controls) | 6/10 | 9/10 | 10/10 | 10/10 | 10/10 |

* Spleen cells and tumour cells mixed and injected together s.c.
† No. tumour takes/No. animals receiving tumour cells.

to the density of antigenic determinants on the tumour-cell surface, to a rapid turnover of antigen or to sequestration of antigen, which might allow the tumour to escape from immunological cytotoxic reactions directed against the antigen. Whilst immunization with EF or CaBP does not appear to induce transplantation-type immunity to the SV40 tumour in the assay systems employed in the present study, this should not be taken as conclusive evidence that the delayed hypersensitivity response to EF seen in animals bearing SV40 tumours is not due to an immunological reaction directed against neoantigen(s) on the tumour-cell surface.

### REFERENCES

BASOMBRIO, M. A. & PREHN, R. T. (1972) A search for common antigenicities between embryonic and tumoural tissues. *Medicina B. Aires*, **32**, 42.

CASPARY, E. A. & FIELD, E. J. (1971) Specific lymphocyte sensitization in cancer: Is there a common antigen in human malignant neoplasia? *Br. Med. J.*, ii, 613.

CHISM, S. E., WALLIS, S., BURTON, R. C. & WARNER, N. L. (1976) Analysis of murine oncofetal antigens as tumour associated transplantation antigens. *J. Immunol.*, **117**, 1870.

COATES, A. S. & CARNEGIE, P. R. (1975) Immunological cross reactivity between basic proteins of myelin and cancer. I. Lymphocyte transformation studies in guinea pigs. *Clin. Exp. Immunol.*, **22**, 16.

DICKINSON, J. P., CASPARY, E. A. & FIELD, E. J. (1972) Localization of tumour specific antigen on external surface of plasma membrane. *Nature, New Biol.*, **239**, 181.

FIELD, E. J. & CASPARY, E. A. (1970) Lymphocyte sensitization: an *in vitro* test for cancer? *Lancet*, ii, 1137.

FLAVELL, D. J., GOEPEL, J., POTTER, C. W. & CARR, I. (1978) Cellular immunity to encephalitogenic factor as measured by macrophage migration inhibition during tumour induction and growth. *Br. J. Cancer*, **37**, 818.

FLAVELL, D. J. & POTTER, C. W. (1978) Cellular immunity to encephalitogenic factor in man as measured by the macrophage migration inhibition test: the effects of serum. *Br. J. Cancer*, **37**, 15.

GOLDSTONE, A. H., KERR, I. & IRVINE, W. J. (1973) The macrophage electrophoretic mobility test in cancer. *Clin. Exp. Immunol.*, **14**, 469.

LIGHT, P. A., PREECE, A. W. & WALDRON, H. A. (1975) Studies with the macrophage migration inhibition test in patients with malignant disease. *Clin. Exp. Immunol.*, **22**, 279.

MCDERMOTT, J. R., CASPARY, E. A. & DICKINSON, J. P. (1974) Antigen cross reactivity in the macrophage electrophoretic mobility test. A study using cellular affinity chromatography. *Clin. Exp. Immunol.*, **17**, 103.

PRITCHARD, J. A. V., MOORE, J. L., SUTHERLAND, W. H. & JOSLIN, C. A. F. (1972) Macrophage electrophoretic mobility (MEM) test for malignant disease. An independent confirmation. *Lancet*, ii, 627.

SHELTON, J. B., POTTER, C. W. & CARR, I. (1975) Cellular immunity to myelin basic protein in man and in animal model systems as measured by the macrophage migration inhibition test. *Br. J. Cancer*, **31**, 528.

WINN, H. J. (1961) Immune mechanisms in homotransplantation. II. Quantitative assay of the immunologic activity of lymphoid cells stimulated by tumour homografts. *J. Immunol.*, **86**, 228.